# COVID-19 outbreak improves attractiveness of medical careers in Chinese senior high school students

**DOI:** 10.1186/s12909-022-03309-7

**Published:** 2022-04-04

**Authors:** Ruoxin Zhang, Jianfeng Pei, Yanli Wang, Lei Wang, Yeerzhati Yeerjiang, Haifeng Gao, Wanghong Xu

**Affiliations:** 1 Department of Epidemiology, Key Lab of Health Technology Assessment (National Health Commission), Key Laboratory of Public Health Safety, Ministry of Education, School of Public Health, Fudan University, 138 Yi Xue Yuan Road, Shanghai, 200032 China; 2 Wuhan No.4 High School, 347 Jie Fang Avenue, Wuhan, 430022 Hubei Province China; 3 Admissions Office, Shanghai Medical College of Fudan University, 138 Yi Xue Yuan Road, Shanghai, 200032 China

**Keywords:** COVID-19, Senior high school students, Medical study, Parents, Motivation

## Abstract

**Background:**

The shortage of healthcare workers is becoming a serious global problem. The underlying reasons may be specific to the healthcare system in each country. Over the past decade, medicine has become an increasingly unpopular profession in China due to the heavy workload, long-term training, and inherent risks. The ongoing COVID-19 pandemic has placed the life-saving roles of healthcare professionals under the spotlight. This public health crisis may have a profound impact on career choices in Chinese population.

**Methods:**

We conducted a questionnaire-based online survey among 21,085 senior high school students and 21,009 parents from 24 provinces (or municipalities) of China. We investigated the change of interest in medical study due to the outbreak of COVID-19 and the potential motivational factors based on the expectancy-value theory framework. Pearson correlation analysis was used to assess the correlation of static or dynamic interest in medical career pursuit with the reported number of COVID-19 cases. Logistic regression model was adopted to analyze the main factors associated with students’ choices.

**Results:**

We observed an increased preference for medical study post the outbreak of COVID-19 in both students (17.5 to 29.6%) and parents (37.1 to 47.3%). Attainment value was found to be the main reason for the choice among students, with the contribution to society rated as the top motivation. On the other hand, the predominant demotivation in high school students was lack of interest, followed by concerns regarding violence against doctors, heavy workload, long-term training and heavy responsibility as a doctor. Additionally, students who were female, in the resit of final year, had highly educated parents and outside of Hubei province were significantly associated with a keen interest in pursuing medical study.

**Conclusions:**

This is the first multi-center cross-sectional study exploring the positive change and motivations of students’ preferences in medical study due to the outbreak of COVID-19. Our results may help medical educators, researchers and policymakers to restructure medical education to make it more appealing to high school students, particularly, to develop a more supportive social and working environment for medical professionals to maintain the observed enhanced enthusiasm.

**Supplementary Information:**

The online version contains supplementary material available at 10.1186/s12909-022-03309-7.

## Introduction

The outbreak of “coronavirus disease 2019” (COVID-19) has overwhelmed the healthcare system in many countries, highlighting the problem of the healthcare worker shortage globally, particularly in China, a country facing exacerbated declining number of doctors during the past decade. Medicine in China is no longer perceived as a notable career as it was in history [1], nor as it is in other countries [2]. Due to the commercialization of health services and limited investment of the Chinese government in healthcare, Chinese medical staff have been overworked and underpaid, and were not fully trusted and respected. The increasing violence against doctors and the reform of China’s medical education system not only have demotivated high school students [3, 4], medical students and young doctors to pursue a career in medicine [5], but also discouraged parents with medical background to support their children to study medicine [6, 7]. A recent survey revealed a dramatic decrease of young doctors from 2005 to 2014, which poses a severe concern for society with rapid population ageing and emerging infectious diseases [5]. It is urgent to encourage the whole society, including high school students, to enhance motivation towards practicing medicine.

The reasons why students seek certain careers are complex. The expectancy-value theory developed by Eccles et al. [8, 9] proposes that achievement-related choices can be predicted by the expectations of success and subjective task values. Namely, students are more likely to pursue careers in which they think they can excel or that have a high value for them [10, 11]. The theory links individual differences in motivational beliefs to their experiences within the school, peer, family, and social contexts, and provides a comprehensive theoretical framework for defining the psychological and contextual factors [8, 9]. The expectancy-value theory has been applied to understand individual choices in Science, Technology, Engineering and Mathematics (STEM) careers [12] and was used to develop effective interventions [13]. It also can be adopted to predict the potential impact of the COVID-19 outbreak on the choices of medical career among Chinese high school students and to identify motivating factors.

Since the outbreak of COVID-19, the world has witnessed unprecedented collective efforts and strengths of the character of many health workers. The roles of doctors, community healthcare workers and nurses have become even more essential and visible in the current epidemic, being defined as “essential workers” by countries. Particularly in China, thousands of healthcare workers across the country joined the frontline workforce in Wuhan of Hubei Province, a city suffering the most since the outbreak of COVID-19. The devotion of healthcare workers gained respect from the whole society and significantly improved the doctor-patient relationship [14–16]. The improved status and image of healthcare staff have significantly reduced the existing external or societal barriers for students to pursue medical career, and therefore increased students’ expectations of success which has been suggested to depend on confidence to overcome possible external or societal barriers to their success [17, 18].

**Fig. 1:**
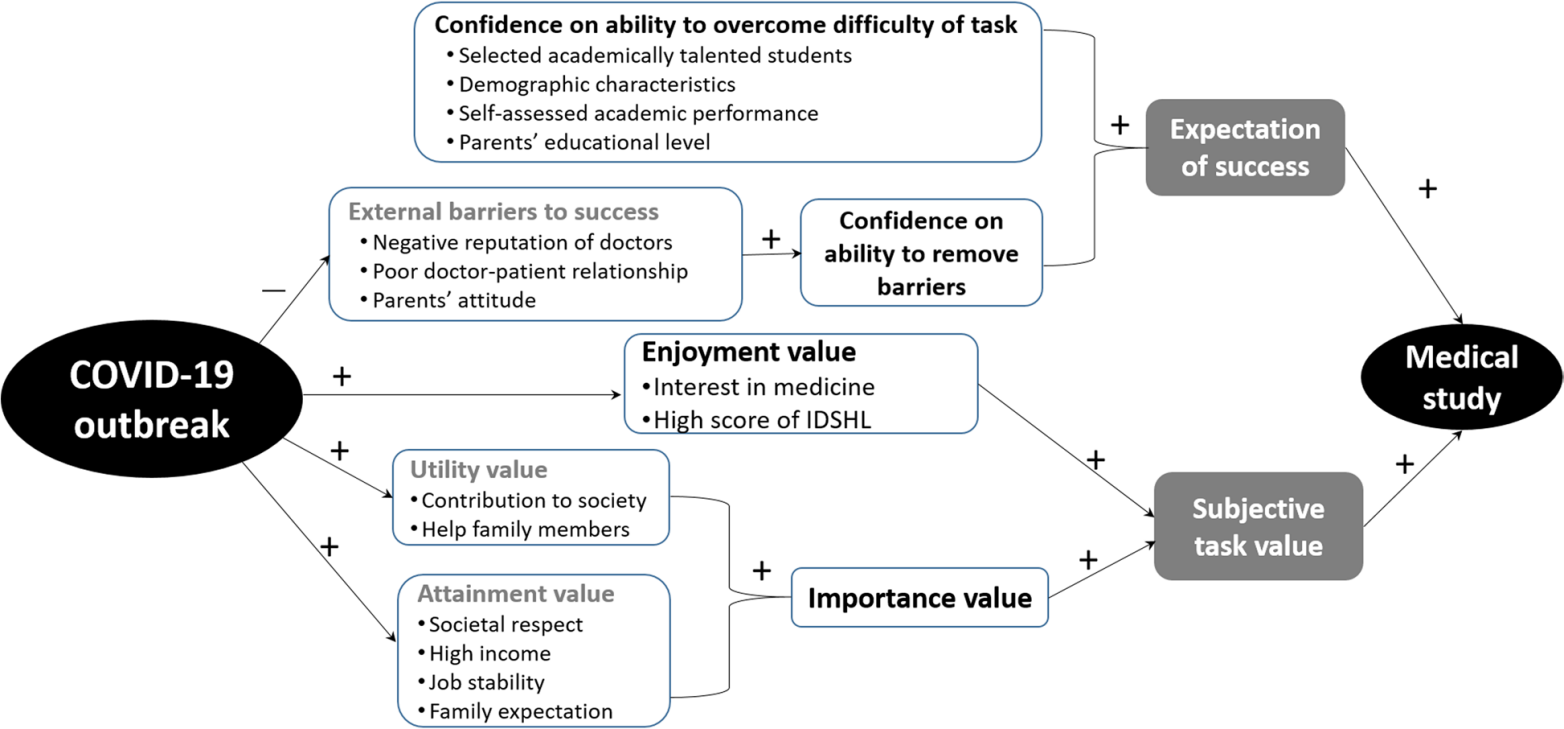
Selected constructs of the expectancy-value model for medical career preference in Chinese senior high school students

Moreover, the severity of COVID-19 may have stimulated motivations in the whole society to defeat and control the infectious disease [13]. This may also increase subjective task values attached to medical study in Chinese students. According to the expectancy-value theory, the subjective task values are assigned to a task based on personal enjoyment (intrinsic interest) and importance values (attainment value and utility value) [18–20]. The enjoyment value is gained from doing the task (in our case the interest in practicing medicine which is intrinsic and partly manifested by high level of health literacy), while the importance value originates from doing well at a given task (the attainment value) and how the task fits into one’s future plans (the utility value), in our case, pursuing medical careers (the task) to defeat severe diseases like COVID-19 (the plans). The constructs related to the domains of the expectancy-value model are presented in Fig. 1. The difficulty of the task (pursuing medical careers) for a high school student is determined by admittance to a medical school first at all. Confidence in the ability to overcome the difficulty is largely dependent on students’ self-evaluated academic performance. Confidence on the ability to remove barriers, on the other hand, relies on the strength of external barriers, i.e. the existing negative attitudes towards medical careers in societies and families in our scenario. The improved social status of medical professionals during the outbreak of COVID-19 may have significantly reduced the barriers and enhanced students’ confidence in their ability to remove the barriers.

This study, therefore, was designed to focus on a portion of the model among students in key senior high schools who might have had confidence on intellectual ability (69.7% of whom had academic performance qualified for top-tier universities and high potential to be admitted to medical schools) and on their ability to remove the decreased external barriers. Specifically, this study aimed to evaluate how improved subjective task values and increased expectation of success due to the outbreak of COVID-19 change the preferences of medical careers in the population.

In current literature, many studies have focused on the career path of existing medical students [13, 21–23], but very few investigated the determinants for the pursuit of medical study in high school students. In a qualitative study based on self-determination theory, Wouters, et al. [24] found that the main reasons for pursuing a medical career in Dutch high school students were pertained to autonomous motivation (interest in science and helping people) and controlled motivation (e.g., parental pressure and prestige). In cross-sectional studies conducted on Chinese senior high school students, sex, grade, medical interest, and family background have been associated with the choices of medical careers [3, 4], all of which are within the domains of the expectancy-value theory [13, 23, 24]. Moreover, several studies have addressed the motivations stimulated by exceptional circumstances [15, 21, 24, 25], but none of them addressed the potential positive impact of pandemic disease on medical career preference in students at the time of high school.

In this study, we analyzed the data derived from an online questionnaire-based survey in China to evaluate impact of the outbreak on attitudes toward medical study in students from top senior high schools and their parents, and to understand the motivating factors for renewed interest on medical career based on framework of expectancy-value theory. Our results may help to identify factors that motivate the choice of medical careers, and thus provide policy-making evidence to relieve the shortage of medical professionals in China.

## Methods

### Study population and procedure

We designed a student-version and a parent-version of self-administered questionnaires (presented as uploaded files at https://data.mendeley.com/datasets/zpvfwnp9cp/3) using the online survey tool Sojump (Shanghai Information Co.), and released the electronic versions by WeChat platform to senior high school students and their parents following a principal -- headteacher -- students/parents approach during the period of 26 February and 4 March 2020. Specificity, we forwarded the online questionnaires to principals of key senior high schools whose WeChat were available by the Admissions Office of the Fudan University first. And then, the principals forwarded the questionnaires to headteachers of all classes in their respective schools. Finally, headteachers released the students’ and parents’ versions of questionnaires to the corresponding WeChat groups consisting of students or parents only. In the parents’ group, only one guardian (mainly father or mother) was included for each student. In rare cases when neither of the parents was available, one grandparent or an other family member would be included as the guardian of the student. All students were at the age of 16–19 years, so consent to participate from their parents / guardians was not needed. All the students and the parents were asked to answer the questions anonymously after reading informed consent at the fore page of the questionnaires. The participant was not required to provide written informed consent. Once clicking the “start” button, he /she was assumed to have read the information about the survey, and voluntarily agreed to participate the study. The principals and the headteachers were also encouraged to forward the online questionnaires to their colleagues in other key senior high schools.

The online questionnaires could be submitted only after all the questions were answered, so there was no missing value in the data collected. Duplicate questionnaires were removed based on IP address to ensure only one questionnaire was completed by one person.

This study was approved by the Institutional Review Board of the Fudan University School of Public Health (IRB00002408 & FWA00002399), and followed the ethical principles of the Declaration of Helsinki 1964.

### Measures and instruments

#### Questionnaire-based information

The questionnaire was designed to collect demographic data, preferences for medicine-related degrees, and potential motivational factors. Students’ questionnaire included sex, name of high school, academic year, performance level, educational level and occupation of parents, degree preferences including willingness to learn medicine (prior and post COVID-19 outbreak), preferred medical career (clinician, public health practitioner, pharmacist, nurses, or others), and main motivations for selecting or unselecting medical study. The question regarding the preference before the COVID outbreak was designed as “Before the COVID outbreak, did you have a plan to apply for medical school?” An Infectious Disease-Specific Health Literacy Scale (IDSHL) developed by *Tian,* et al. [26] was adopted to assess student’s health literacy.

The questions in medical study mainly focused on the impact of COVID-related and demographic variables, which were widely discussed with teachers and principals from participating high schools. Framed by expectancy-value theory, a singular choice question was designed to identify the driving motivations for medical study. All students willing to learn medicine were asked to select one out of the eight potential motivations: 1) interest in medicine; 2) contribution to society; 3) societal respect; 4) high income; 5) job stability; 6) helping family members; 7) family expectation and 8) others. If “others” was selected, further details were required. As shown in Supplementary Table 1, these motivating factors could be categorized into domains of enjoyment value and importance value (i.e., attainment value and utility value) according to the expectancy-value theory. For those who didn’t select medical studies, they were asked to select main concerns from the 10 choices: 1) lack of interest; 2) heavy workload; 3) high responsibility; 4) high risk of infection; 5) violence against doctors; 6) low income; 7) long/difficult training; 8) crowded environment; 9) low family expectation, and 10) others. The details were required if “others” was selected.

Information collected from parents included sex, name of high school attended by their children, educational level, occupation, attitude toward their child studying medicine, and main reasons for supportive or unsupportive attitudes which were similar to the main motivations or demotivations for medical study listed in the student-version questionnaire. The date and time for completion of the questionnaire were auto-recorded by the Sojump system. As a quality control procedure, all observations that did not provide detailed names of the senior high schools were excluded for possible inaccurate data. Due to the anonymous distribution of the questionnaires, the respondents of the students’ questionnaire could only be matched with those of the parents at the population level, but not at the individual level. The dataset can be accessed through the website link of https://data.mendeley.com/datasets/zpvfwnp9cp/3.

#### The IDSHL

The IDSHL was developed, tested and validated in the Chinese population in 2014 [26], and was used to assess health literacy level of the students on infectious diseases in this study. The self-reported scale includes 28 items, with a total score ranging from 0 to 100. A higher score corresponds to a higher level of health literacy. Of the 28 items, 22 were scored according to the difficulty level of the questions and were classified into 4 domains: 1) infectious disease-related knowledge and values; 2) prevention of infectious diseases; 3) management or treatment of infectious diseases; and 4) identification of pathogens and infection sources. In this study, the IDSHL demonstrated good internal consistency (Cronbach’s α = 0.758).

#### COVID-19 epidemic data

The number of COVID-19 cases across China was obtained through the website of the National Health Commission of the People’s Republic of China (http://www.nhc.gov.cn/xcs/xxgzbd/gzbd_index.shtml). The data accessed included the number of daily reported new cases regional and national (26th February - 4th March 2020) and the number of accumulated cases in the participants’ regions (provinces, municipalities, and autonomous regions) and around the country (until 4th March 2020). The percentage of regional cases of COVID-19 was calculated as regional accumulated cases divided by the total number of accumulated cases. The key events that occurred in the early period of COVID-19 pandemic were also derived from the COVID-19 Timeline Report of the WHO and the National Health Commission of the People’s Republic of China.

### Statistical analysis

With the sample size of 21,085 students and 21,009 parents, the power was 100% for estimating the changes in attitudes toward medical study and identifying potential influence factors.

The characteristics of the students and their parents were summarized, respectively. The number and proportion of participating students who had positive attitude toward medical career and the number and proportion of supportive parents were stratified by sex, parents’ education level, medical background, geographic region, academic year, academic performance, and IDSHL score. McNemar test was used to compare the difference in percentages between paired groups. Interquartile range (IQR) for each subgroup was calculated from the schools with more than 100 participating students, and quartile 1 and quartile 3 were presented. The regional or time correlation of medical degrees selection and number of COVID-19 cases were illustrated in diagrams and evaluated using Pearson or Spearmen’s correlation analyses. Hubei province data was censored only in the correlation analysis due to its relatively small sample size in comparison with data from other provinces. For logistic regression analysis, both univariate and multivariate analysis were performed to assess the main factors associated with selection of medical study. A *P* value of < 0.05 was considered statistically significant. All the data were analyzed by SAS (v9.4) or R (v3.6.1) software.

## Results

### Characteristics of the study population

From 42,557 surveys that were delivered, we excluded 56 students and 20 parents due to incomplete names of high schools and provinces and thus unclear locations. We also removed 392 grandparents from the analyses. The effective numbers of participants were 21,085 students and 21,009 parents from 233 senior high schools in 24 provinces, municipalities and autonomous regions (96 cities), in which 776 students and 802 parents were from Wuhan, the capital city of Hubei Province suffering the most from the COVID-19 outbreak. The geographic distribution of the participants is illustrated in Supplementary Fig. 1.

The demographic characteristics and medical study preferences of the sampled students are shown in Table 1. Of the respondent students, 52.9% were females, 3.8% came from Hubei province. The distribution of students in Year 1, Year 2, graduate year, and resit graduate year were 33.4, 31.8, 33.1, and 1.8%, respectively. According to student self-evaluation, 69.7% of the students were predicted to have academic performance qualified for top-tier universities. The median IDSHL score of the students was 73 (out of 100). Only 1.6% of the students claimed to have had acquaintance infected with COVID-19. With regards to parents’ education level, 21.9% of fathers and 17.6% of mothers were reported with a degree of university diploma or above (Table 1), comparable to the overall 27.9% of the highest degree self-reported by parents (Supplementary Table 2). In the parents’ survey, 4.9% of the parents indicated their background in healthcare.

**Table 1:** Characteristics of student participants and their interest in pursuing medical study prior and post the COVID-19 outbreak

Characteristics	Total (%)^**a**^	Before COVID-19			After COVID-19			Difference^**e**^	χ^**2**^	*P value* ^***f***^
		No^**b**^	Percentage^**c**^	IQR^**d**^	No^**b**^	Percentage^**c**^	IQR^**d**^			
All subjects	21,085	3682	17.5	(13.9, 20.9)	6249	29.6	(23.6, 35.6)	12.1	*2175.5*	*< 0.001*
Sex										
Male	9933 (47.1)	1583	15.9	(11.4, 19.7)	2658	26.8	(20.6, 31.2)	10.9	*907.8*	*< 0.001*
Female	11,152 (52.9)	2099	18.8	(15.7, 22.3)	3591	32.2	(27.5, 38.4)	13.4	*1267.7*	*< 0.001*
Father’s education										
Below primary school	1727 (8.2)	276	16.0	(10.7, 21.3)	538	31.2	(18.3, 43.8)	15.2	*235.1*	*< 0.001*
Junior school	5831 (27.7)	962	16.5	(12.0, 20.4)	1767	30.3	(24.6, 35.8)	13.8	*696.1*	*< 0.001*
High school	5934 (28.1)	1060	17.9	(13.2, 20.7)	1774	29.9	(24.2, 35.0)	12.0	*609.8*	*< 0.001*
Diploma	2972 (14.1)	534	18.0	(12.8, 22.3)	900	30.3	(23.6, 38.0)	12.3	*318.9*	*< 0.001*
University or above	4621 (21.9)	850	18.4	(13.4, 25.6)	1270	27.5	(23.0, 34.4)	9.1	*320.7*	*< 0.001*
Mother’s education										
Below primary school	2868 (13.6)	446	15.6	(12.3, 23.6)	886	30.9	(24.8, 39.2)	15.3	*400.0*	*< 0.001*
Junior school	6234 (29.6)	1061	17.0	(12.7, 19.7)	1886	30.3	(23.2, 34.6)	13.3	*706.8*	*< 0.001*
High school	4933 (23.4)	853	17.3	(10.6, 22.0)	1443	29.3	(21.2, 36.3)	12.0	*504.5*	*< 0.001*
Diploma	3339 (15.8)	641	19.2	(11.2, 22.5)	1007	30.2	(21.7, 35.3)	11.0	*299.0*	*< 0.001*
University and above	3711 (17.6)	681	18.4	(16.6, 28.2)	1027	27.7	(23.8, 39.5)	9.3	*269.6*	*< 0.001*
Region										
Hubei	809 (3.8)	100	12.4		167	20.6		8.2	*50.4*	*< 0.001*
Non-Hubei	20,276 (96.2)	3582	17.7	(14.4, 21.1)	6082	30.0	(24.0, 35.7)	12.3	*2125.9*	*< 0.001*
Academic year										
Year 1	7032 (33.4)	1193	17.0	(13.5, 19.7)	2155	30.6	(24.2, 34.8)	13.6	*845.9*	*< 0.001*
Year 2	6698 (31.8)	1019	15.2	(10.6, 18.8)	1789	26.7	(19.8, 35.3)	11.5	*651.5*	*< 0.001*
Graduate year	6984 (33.1)	1373	19.7	(12.9, 20.6)	2158	30.9	(22.9, 35.3)	11.2	*639.9*	*< 0.001*
Resit of graduate year	371 (1.8)	97	26.1	(0, 30.8)	147	39.6	(9.1, 100.0)	13.5	*40.3*	*< 0.001*
Academic performance										
Top tier	14,698 (69.7)	2695	18.3	(14.3, 21.7)	4352	29.6	(24.0, 34.8)	11.3	*1390.2*	*< 0.001*
Second tier	4679 (22.2)	733	15.7	(10.8, 20.0)	1402	30.0	(21.9, 36.6)	14.3	*576.0*	*< 0.001*
Third tier	687 (3.3)	117	17.0	(0, 20.0)	239	34.8	(25.0, 46.6)	17.8	*112.8*	*< 0.001*
Others	1021 (4.8)	137	13.4	(0.0, 21.1)	256	25.1	(6.3, 37.5)	11.7	*97.7*	*< 0.001*
Any acquaintance with COVID-19										
Yes	328 (1.6)	49	14.9	(0, 33.3)	82	25.0	(0, 50.0)	10.1	*26.6*	*0.0013*
No	20,757 (98.4)	3633	17.5	(14.0, 20.9)	6167	29.7	(23.5, 35.8)	12.2	*2149.0*	*< 0.001*
IDSHL score										
≤ 73	10,759 (51.0)	1500	13.9	(10.5, 16.5)	2761	25.6	(19.8, 31.0)	11.7	*1056.6*	*< 0.001*
>73	10,326 (49.0)	2182	21.2	(16.8, 25.5)	3488	33.8	(27.7, 39.1)	12.6	*1119.1*	*< 0.001*

^a^Total number of students in all or subgroups and percentage of the subgroup

^b^Number of students who selected medicine

^c^Percentage referring to the percentage of students who selected medicine in the subgroup, each value represents the percentage

^d^*IQR:* Interquartile range, represented by the Q1 and Q3 value from the 36 schools with more than 100 participants

^e^Difference calculated as the subtraction the percentage of students selecting medicine during COVID-19 from the percentage before the outbreak

^f^χ^2^ and *P* values for McNemar tests in each subgroup

### Increased preference for medical study since COVID-19

Overall, the percentage of students who selected medical study increased significantly from 17.5% (IQR: 13.9 to 20.9%) to 29.6% (IQR: 23.6 to 35.6%) before and after the COVID-19 outbreak (*P* <  0.0001, Table 1). A similar trend was observed across different subgroups. Female students seemed to have a growing positive attitude toward studying medicine after the outbreak, compared to male students (percentage change: 13.4% vs. 10.9%). Despite the relative smaller sample size of Hubei participants, a lower increase in preference towards medical study was observed in these students, compared with those outside of Hubei province (percentage change: 8.2% vs. 12.3%). Regarding the infectious disease literacy level, students with a higher score of IDSHL (> 73) showed a higher growth of interest in medical study, compared to those below the median score (percentage change: 12.6% vs. 11.7%).

Parents’ results showed a more positive attitude towards favoring their children studying medicine than the students themselves. The outbreak of COVID-19 increased the overall proportion of supportive parents from 37.1% (IQR: 29.7 to 40.8%) to 47.3% (37.5 to 54.6%). The increase was more evident in non-medical professional parents (from 36.7 to 47.1%) than in medical professional parents (from 44.1 to 49.4%) (Supplementary Table 2).

### Correlation of interest in medicine and epidemic status of COVID-19

**Fig. 2:**
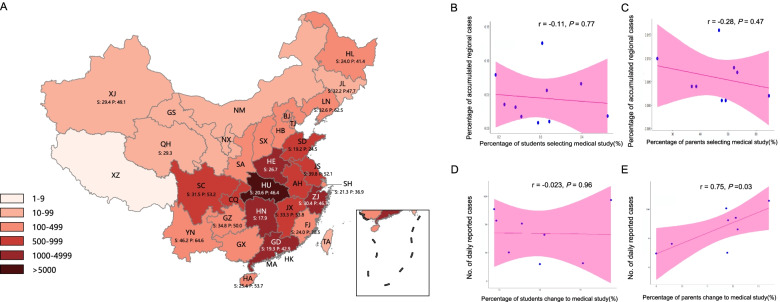
Preference toward medical study and epidemic status of COVID-19 across regions in China. **A**. Distribution of the cumulative number of COVID-19 cases (up to March 4th 2020) and percentage of students (S) or parents (P) selecting medical study; **B**. correlation of the percentage of students selecting medical studies and the percentage of accumulated positive COVID-19 case in each of 10 provinces accounting for the national accumulated cases; **C**. correlation of the percentage of parents selecting medical study and the percentage of accumulated positive COVID-19 case in each of 9 provinces accounting for the national accumulated cases; **D.** correlation of the percentage of students changed to selecting medical study and the number of daily reported new cases across 10 provinces; **E.** correlation of the percentage of parents changed to selecting medical study and the number of daily reported new cases across 9 provinces

The post hoc preference towards medicine was mapped against the accumulated number of reported COVID-19 cases until 4th March of 2020 by regions (Fig. 2A). Correlation analyses were further performed in both students and parents to investigate the relationship between preference towards medicine and COVID-19 severity. In order to establish a more robust correlation, only regions with participant number of more than 100 were included. As shown in Fig. 2B and C, no significant correlation was found between the percentage of medical study selection and the percentage of accumulated regional cases over the study period in both the students (*r* = − 0.11, *P* = 0.77) and parents (*r* = − 0.28, *P* = 0.47). However, a potential negative correlation was indicated across regions in both datasets.

Correlation analysis was also conducted between the decision change (from no selection or indifference to the selection of medical study) and daily reported cases in both students (Fig. 2D) and parents (Fig. 2E). In the parents’ dataset, a significant positive correlation was observed between the number of daily COVID-19 cases and the change of medical study selection (*r* = 0.75, *P* = 0.03), which indicates that the interest of parents grew along with the rise of daily reported COVID-19 cases. Other analyses revealed no significant correlations with the current sample size.

### Factors associated with medical study preference

In order to evaluate the variables that may affect students’ choice of medical study, logistic regression analysis was performed. As shown in Table 2, in univariate analysis, students who were female, in the resit of graduation year, based outside of Hubei province and with higher IDSHL score were significantly associated with selection of medical study (*P*_uni_ <  0.001, *P*_FDR_ < 0.0001). The educational level of parents seemed to pose an opposite effect, as parents with diploma or university degrees were associated with a decreased percentage of students favoring medical study. Students in the second year of high school also showed a decreased interest in medical study. However, after multivariate adjustment, the degree of significance for parents’ educational level changed (*P*_adj_ = 0.042 for fathers with diploma or above and *P*_adj_ = 0.270 for mothers with diploma or above), whereas the rest remained similar.

**Table 2:** Logistic regression analysis for underlying factors associated with medical study in the students’ dataset

Characteristics	No.	Univariate analysis					Multivariate analysis				
		β	SE	OR (95%CI)	***P***	***P***_**FDR**_^**c**^	β	SE	OR (95%CI)	***P***_**adj**_^**a**^	***P***_**FDR**_
Sex											
Male	9933	Ref		Ref			Ref		Ref		
Female	11,152	0.260	0.030	1.30 (1.22–1.38)	*< 0.0001*	*< 0.0001*	0.254	0.03	1.29 (1.22–1.37)	*< 0.0001*	*< 0.0001*
Father’s education											
Below high school	7558	Ref		Ref			Ref		Ref		
High school	5934	−0.030	0.040	0.97 (0.90–1.05)	*0.450*		− 0.013	0.04	0.99 (0.91–1.07)	*0.757*	
Diploma or above	7593	−0.090	0.040	0.91 (0.85–0.98)	*0.0096*	*0.021*	−0.100	0.05	0.90 (0.82–1.00)	*0.042*	*0.107*
Mother’s education											
Below high school	9102	Ref		Ref			Ref		Ref		
High school	4933	−0.060	0.040	0.94 (0.88–1.02)	*0.138*		−0.034	0.04	0.97 (0.89–1.05)	*0.444*	
Diploma or above	7050	−0.080	0.030	0.93 (0.86–0.99)	*0.027*	*0.050*	−0.055	0.05	0.95 (0.86–1.04)	*0.270*	
Region											
Hubei	809	Ref		Ref			Ref		Ref		
Outside of Hubei	20,276	0.500	0.090	1.65 (1.39–1.96)	*< 0.0001*	*< 0.0001*	0.270	0.09	1.31 (1.10–1.56)	*< 0.0001*	*< 0.0001*
Academic year											
First year	7032	Ref		Ref			Ref		Ref		
Second year	6698	0.193	0.040	0.82 (0.77–0.89)	*< 0.0001*	*< 0.0001*	−0.199	0.10	0.82 (0.67–1.00)	*< 0.0001*	*< 0.0001*
Graduate year	6984	0.012	0.040	1.01 (0.94–1.09)	*0.745*		−0.004	0.04	1.00 (0.92–1.08)	*0.919*	
Resit of graduate year	371	0.396	0.110	1.49 (1.20–1.84)	*0.0003*	*< 0.0001*	0.349	0.04	1.42 (1.31–1.53)	*0.002*	*0.004*
Academic performance											
Top tier	4698	Ref		Ref			Ref		Ref		
Second tier	4679	0.017	0.037	1.02 (0.95–1.09)	*0.644*		0.025	0.039	1.03 (0.95–1.11)	*0.514*	
Third tier and others	1708	0.030	0.056	0.97 (0.87–1.08)	*0.590*		0.009	0.059	1.00 (0.90–1.13)	*0.876*	
Any acquaintance with COVID-19											
No	20,757	Ref		Ref			Ref		Ref		
Yes	328	0.237	0.128	0.79 (0.61–1.01)	*0.064*		0.059	0.14	1.06 (0.81–1.40)	*0.670*	
IDSHL score^b^											
≤73	10,759	Ref		Ref			Ref		Ref		
>73	10,326	0.395	0.030	1.48 (1.40–1.57)	*< 0.0001*	*< 0.0001*	0.418	0.03	1.52 (1.43–1.61)	*< 0.0001*	*< 0.0001*

^a^Adjusted by all the variables as listed in the characteristics

^b^*IDSHL:* Infectious disease-specific health literacy

^c^The adjusted *P* value based on Benjamini-Hochberg FDR test

Among the selected subjects related to medical study, clinical medicine was the most popular (54.5%), followed by Chinese traditional medicine (14.1%), pharmacy (6.9%), Chinese pharmacy (5.6%), public health (3.7%), and nursing (2.6%) (Supplementary Fig. 2).

### Predominant motivators for students to select medical study

**Fig. 3:**
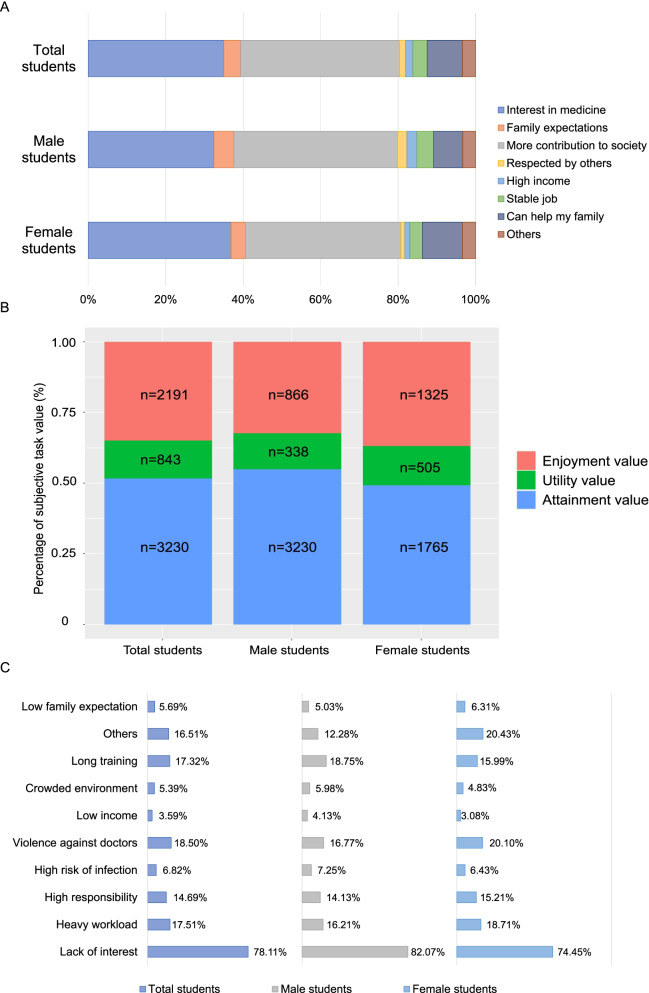
Motivational and de-motivational factors to select medical study in Chinese senior high school students. **A**. distribution of motivations for medical study; **B**. Distribution of motivation domains based on expectancy-value model; C. distribution of de-motivations for medical study (%)

In order to further investigate the underlying motivations for the change of interest in medicine, the reasons for selecting medicine were assessed in the students’ datasets. Contribution to society ranked as the top reason in the dataset, followed by interest in medicine. Interestingly, family expectation accounted for 4.4% of motivations in students (Fig. 3A). When the motivations were further categorized according to the expectancy value theory, the predominant motivations were found to account for the attainment value (52%), followed by enjoyment and utility values (35 and 13%, respectively, Fig. 3B).

On the other hand, demotivating factors were multi-faceted. Based on the nature of multiple-choice questions, the predominant demotivations in high school students were lack of interest, followed by concerns regarding violence against doctor, heavy workload, long-term training and heavy responsibility for doctors (Fig. 3C).

## Discussion

The world is experiencing a shortage of healthcare professionals, especially in China over the past 10 years [27]. The most severe shortage was seen in pediatricians, general practitioners, and psychiatrists [28]. Although medical education admission has been expanded since 1998 in China, the problems with workforce in healthcare and the reasons for reduced interest in pursuing medical careers have not been fully addressed. The most common reasons include low income for trainee doctors and deterioration of doctor-patient relations [25, 29]. The expansion of medical programs, to meet an increased demand, may in turn reduce the quality of healthcare due to lower admission standards for medical students. The additional 2–4 year training for junior doctors introduced by China has raised doubts about the delayed career development for young doctors, which further demotivated students to embark on a long medical career [30].

In this study, a significant increasing trend in positive attitudes towards medical study was observed from both high school students and their parents after the outbreak of COVID-19. The increase of interest was observed in all subgroups and across the nation. Therefore, it can be anticipated that more top-tier students in China are willing to select medicine as a future career to tackle the pressing issue of medical worker shortage. More importantly, this study advances our understanding of the influence of the outbreak of COVID-19 in improving students’ preferences for medical study by exploring the change in the constructs and their associations with medical study based on the expectancy-value model. Our findings indicate that the optimal way to encourage high school students to pursue career in medicine is to substantially improve social status and images of Chinese medical professionals, an occupation featured by fierce competition in admission, more challenge and pressure in work, increasing conflicts with patients, and unattractive salaries [3].

Sex, region, academic year, fathers’ education, and infectious disease literacy were found to be significant predictors for the choice of medical study in students. All these factors may be relevant to the students’ confidence on their abilities to overcome difficulty of medical study and potential external barriers to success (namely, expectation of success). Interestingly, we did not observe a significant association of self-reported academic performance with preference of medical career, appearing to be inconsistent with the expectancy value theory (*P*_adj_ = 0.514 for “second tier” and 0.876 for “third tier and others”). However, this study just focused on the students and their parents from key senior high schools where the students were admitted by their excellent academic performance. The students might be more homogeneous, minimizing the potential influence of school and peer contexts. Infectious disease literacy may also indicate intrinsic interest in medicine in our subjects.

The underlying motivations for medical career choices, on the other hand, were predominantly within the importance value of the expectancy value theory, which reflects the public focus on healthcare in this unusual time. Even though motivations for enjoyment value (Interest in medicine: 35.0%) were regarded as the most important for students’ achievement outcomes and positive well-being, factors within importance value are shown to be the predominant motivators in similar studies carried out in upper-middle income countries [29, 31]. In the degree choice results, the majority of students expressed interest in clinical medicine, with public health ranks the 6th on the list of healthcare-related degrees. This indicates that this outbreak has not raised interest in this important domain as much as anticipated. However, given the number of Chinese healthcare professionals for disease prevention and control is only about one-fifth of that in the United States [32], the demands for more workforce in public health may remain high over the next a few years.

The explanations for the results by nature are multifactorial, probably including specific sociocultural, contextual, biological, and psychological factors that shape individual’s motivational belief according to the expectancy-value theory [9]. Although the positive change in medical career perception in Chinese students and parents could not be directly linked to COVID-19, it can be extrapolated that the changes might be inspired by several COVID-19-related factors. First, the performance of Chinese medical staff during the COVID-19 outbreak has successfully restored their positive reputation in society, greatly reducing the external or social barriers to learning medicine and success. Second, the beliefs that doctors are healers with benevolent hearts are still widely held in society, including in the young generation, which was consistent with findings from Wang, et al [28], and may contribute to the increased utility value and attainment value of practicing medicine. Third, the interest in medical study may be stimulated by the severity of COVID-19, greatly improving enjoyment value in the students. From our correlation analysis, the dynamic change of preference may be linked with reported COVID-19 cases, indicating the possible motivation in Chinese students to combat with the disease.

The strengths of the study include the large sample size, the timing of investigation, the wide distribution of the study subjects, the detailed information collected, and intensive analysis of the data. Particularly, the questions on the potential motivating factors for medical study were designed based on the expectancy-value theory, deepening our understanding of how COVID-19 pandemic improved motivations to study medicine in our subjects. Moreover, we followed a principal -- head teacher -- students/parents approach to release the online questionnaire through WeChat platform to the class groups (including students only) and the corresponding parent groups, respectively. The head teacher of each class was in both groups, which may have minimized data entry by participants apart from the study population.

The major limitation of this study is the use of snowball sampling method, which may have led to selection bias. It is possible that students and parents who were interested in medical study were more likely to attend the survey, resulting in overestimation on preference in medical study. Moreover, due to the fact that the online questionnaires were submitted only after all questions were answered, we could not obtain a non-response rate and evaluate selection bias. However, the large sample size and the small variation in the percentage of interest across different high schools in the country partly released our concern. Second, the questionnaire was anonymous, which means possible recall or report bias, particularly on the willingness to choose medical study before the COVID-19 outbreak. The anonymous and separate delivery of the questionnaires make it impossible to match the student participants with the parent participants individually; therefore the number of students and parents may not be equal in the subgroups. Nevertheless, as the online questionnaires were released to the WeChat groups of students and the corresponding groups of parents, the responses of the students and the parents can be matched as a whole. Third, our results indicate a significant trend in the change of attitude over a period of time when the number of COVID-19 cases increased rapidly. However, differences may exist between the reporting jurisdictions and the number from the WHO website due to the lagging time of reporting and the update of the website, which may cause subtle change of our results. Furthermore, due to the nature of cross-sectional study, a longitudinal study is required to determine and replicate the choices among our subjects, and confirm the impact of pandemic development of COVID-19 on the attitudes toward medical study.

## Conclusions

In conclusion, this study observes a significantly improved attractiveness of medical career in China due to the outbreak of COVID-19. This study also offers insights to prepare for the impacts of COVID-19 on the perception of medical degrees across different regions in China. Understanding the dynamic change of motivations may help medical educators, researchers and policymakers to restructure medical education to make it more appealing to high school students, particularly, to develop a more supportive social and working environment for medical professionals to maintain the observed enhanced enthusiasm.

## Supplementary Information

**Additional file 1: Supplementary Table 1.** Domains of risk factors and reasons for medical career preference based on the expectancy-value theory. **Supplementary Table 2.** Characteristics of parent participants and their expectations for their children to learn medicine before and after the COVID 19 outbreak. **Supplementary Figure 1.** Geographic distribution of participants across China. **Supplementary Figure 2.** The ranking of medical-related majors preferred by students.

## Data Availability

The datasets used and/or analyzed during the current study are available from the corresponding author on reasonable request.

## Abbreviations

COVID-19: Coronavirus disease 2019
IDSHL: Infectious Disease-Specific Health Literacy Scale
STEM: Science, Technology, Engineering and Mathematics
IQR: Interquartile range
