# Supplementary material for: COVID-19 outbreak improves attractiveness of medical careers in Chinese senior high school students
Source: BMC Med Educ. 2022 Apr 4;22:241. doi: 10.1186/s12909-022-03309-7 (PMC8978502; doi:10.1186/s12909-022-03309-7)
Supplement: Supplementary file 1 — Additional file 1: Supplementary Table 1. Domains of risk factors and reasons for medical career preference based on the expectancy-value theory. Supplementary Table 2. Characteristics of parent participants and their expectations for their children to learn medicine before and after the COVID 19 outbreak. Supplementary Figure 1. Geographic distribution of participants across China. Supplementary Figure 2. The ranking of medical-related majors preferred by students. [file 12909_2022_3309_MOESM1_ESM.docx]

**Supplementary Table 1.** Domains of risk factors and reasons for medical career preference based on the expectancy-value theory

| **Related factors with preference** | |  | **Reasons for preference** | |
| --- | --- | --- | --- | --- |
| **Factors** | **Domains** |  | **Points** | **Domains** |
| Sex | Expectation of success |  | Interest in medicine | Enjoyment value |
| Parents’ education |  |  | Contribution to society | Importance value (utility value) |
| Academic performance |  |  | Helping family members |  |
| Academic year |  |  | Family expectation | Importance value (attainment value) |
| IDSHL score | Enjoyment value |  | Social respect |  |
| Any acquaintance with COVID | Importance value (utility value) |  | High income |  |
| Region (severity of COVID-19) |  |  | Job Stability |  |

| **Supplementary Table 2**. Characteristics of parent participants and their expectations for their children to learn medicine before and after the COVID 19 outbreak. |
| --- |
| \| **Characteristics** \| \| **Total (%)^a^** \| **Before COVID-19** \| \| \|  \| **After COVID-19** \| \| \| **Difference ^e^** \| **χ^2^** \| ***P-value ^f^*** \| \| --- \| --- \| --- \| --- \| --- \| --- \| --- \| --- \| --- \| --- \| --- \| --- \| --- \| \| **No.^b^** \| **Percent.^c^** \| **IQR ^d^** \|  \| **No.^b^** \| **Percent.^c^** \| **IQR** \| \|  \| \| **Total** \|  \| 21009 \| 7789 \| 37.1 \| (29.7, 40.8) \|  \| 9927 \| 47.3 \| (37.5, 54.6) \| 10.2 \| 1615.2 \| *<0.0001* \| \| **Sex** \| **Male** \| 7152 (34.0) \| 2696 \| 37.7 \| (30.1, 42.0) \|  \| 3485 \| 48.7 \| (40.2, 54.2) \| 11.0 \| 615.7 \| *<0.0001* \| \|  \| **Female** \| 13857 (66.0) \| 5093 \| 36.8 \| (30.8, 43.5) \|  \| 6442 \| 46.5 \| (37.2, 54.5) \| 9.7 \| 1000.4 \| *<0.0001* \| \| **Educational level** \| **Uneducated** \| 68 (0.3) \| 23 \| 33.8 \| (0, 66.7) \|  \| 25 \| 36.8 \| (0.0, 66.7) \| 2.9 \| 0.7 \| *0.4142* \| \|  \| **Primary school** \| 1173 (5.6) \| 473 \| 40.3 \| (30.9, 50.8) \|  \| 571 \| 48.7 \| (45.2, 63.4) \| 8.4 \| 73.9 \| *<0.0001* \| \|  \| **Junior school** \| 4588 (21.8) \| 1968 \| 42.9 \| (33.3, 48.9) \|  \| 2545 \| 55.5 \| (41.7, 60.4) \| 12.6 \| 473.6 \| *<0.0001* \| \|  \| **High school** \| 2527 (12.0) \| 1026 \| 40.6 \| (32.8, 45.8) \|  \| 1328 \| 52.6 \| (43.5, 59.7) \| 12.0 \| 237.5 \| *<0.0001* \| \|  \| **Technical school** \| 2537 (12.1) \| 987 \| 38.9 \| (29.3, 48.9) \|  \| 1277 \| 50.3 \| (39.5, 60.0) \| 11.4 \| 238.9 \| *<0.0001* \| \|  \| **Diploma** \| 4263 (20.3) \| 1470 \| 34.5 \| (29.5, 40.5) \|  \| 1894 \| 44.4 \| (36.6, 54.5) \| 9.9 \| 317.6 \| *<0.0001* \| \|  \| **University and above** \| 5853 (27.9) \| 1842 \| 31.5 \| (26.4, 37.5) \|  \| 2287 \| 39.1 \| (31.5, 49.3) \| 7.6 \| 287.4 \| *<0.0001* \| \| **Marital status** \| **Married** \| 19860 (94.5) \| 7376 \| 37.1 \| (29.5, 42.1) \|  \| 9395 \| 47.3 \| (37.8, 55.0) \| 10.2 \| 1523.9 \| *<0.0001* \| \|  \| **Divorce** \| 906 (4.3) \| 320 \| 35.3 \| (25.0, 41.2) \|  \| 414 \| 45.7 \| (33.3, 60.0) \| 10.4 \| 71.3 \| *<0.0001* \| \|  \| **Other** \| 243 (1.2) \| 93 \| 38.3 \| (26.1, 50.0) \|  \| 118 \| 48.6 \| (29.8, 59.6) \| 10.3 \| 20.2 \| *0.0221* \| \| **Sex of child** \| **Male** \| 10785 (51.3) \| 4061 \| 37.7 \| (29.7, 46.3) \|  \| 5221 \| 48.4 \| (38.6, 56.4) \| 10.8 \| 895.9 \| *<0.0001* \| \|  \| **Female** \| 10224 (48.7) \| 3728 \| 36.5 \| (28.3, 41.0) \|  \| 4706 \| 46.0 \| (34.2, 53.0) \| 9.6 \| 720.2 \| *<0.0001* \| \| **Academic year of child** \| **Year 1** \| 6604 (31.4) \| 2482 \| 37.6 \| (30.9, 39.2) \|  \| 3192 \| 48.3 \| (39.6, 54.9) \| 10.8 \| 540.9 \| *<0.0001* \| \|  \| **Year 2** \| 6280 (29.9) \| 2206 \| 35.1 \| (26.2, 40.4) \|  \| 2837 \| 45.2 \| (34.9, 51.6) \| 10.0 \| 488.5 \| *<0.0001* \| \|  \| **Graduate year** \| 7901 (37.6) \| 2997 \| 37.9 \| (30.0, 40.6) \|  \| 3771 \| 47.7 \| (36.5, 54.0) \| 9.8 \| 568.4 \| *<0.0001* \| \|  \| **Resit of graduate year** \| 224 (1.1) \| 104 \| 46.4 \| (27.1, 50.0) \|  \| 127 \| 56.7 \| (37.5,69.7) \| 10.3 \| 18.2 \| *<0.0001* \| \| **Academic** **performance of child** \| **Top-tier** \| 15933 (75.8) \| 5751 \| 36.1 \| (30.4, 40.2) \|  \| 7300 \| 45.8 \| (37.2, 53.1) \| 9.7 \| 1142.0 \| *<0.0001* \| \|  \| **Second tier** \| 3904 (18.6) \| 1582 \| 40.5 \| (32.1, 46.7) \|  \| 2035 \| 52.1 \| (42.5,55.6) \| 11.6 \| 372.4 \| *<0.0001* \| \|  \| **Third tier** \| 507 (2.4) \| 210 \| 41.4 \| (19.4, 49.6) \|  \| 291 \| 57.4 \| (25.5, 68.1) \| 16.0 \| 72.1 \| *<0.0001* \| \|  \| **Others** \| 665 (3.2) \| 246 \| 37.0 \| (25.5. 50.0) \|  \| 301 \| 45.3 \| (30.1, 51.7) \| 8.3 \| 34.8 \| *<0.0001* \| \| **Healthcare background** \| **Yes** \| 1031 (4.9) \| 455 \| 44.1 \| (33.3, 55.6) \|  \| 509 \| 49.4 \| (36.7, 58.3) \| 5.2 \| 42.6 \| *<0.0001* \| \|  \| **No** \| 19978 (95.1) \| 7334 \| 36.7 \| (28.8, 40.2) \|  \| 9418 \| 47.1 \| (37.5, 54.7) \| 10.4 \| 1572.6 \| *<0.0001* \| |
| ^a^ Total number of parents in the subgroup and percentage of the subgroup; ^b^ Number of parents who expects for their children to learn medicine; ^c^ Percent. Referring to the percentage of students who selected medicine in the subgroup, each value represents the percentage; ^d^ IQR: Interquartile range, represented by the Q1 and Q3 value from 34 schools with more than 100 participants. ^e^ Difference as the subtraction the percentage of students selecting medicine during COVID-19 from the percentage before the outbreak. ^f^ **χ^2^** and *P* values for McNemar tests in each subgroup.. |

**
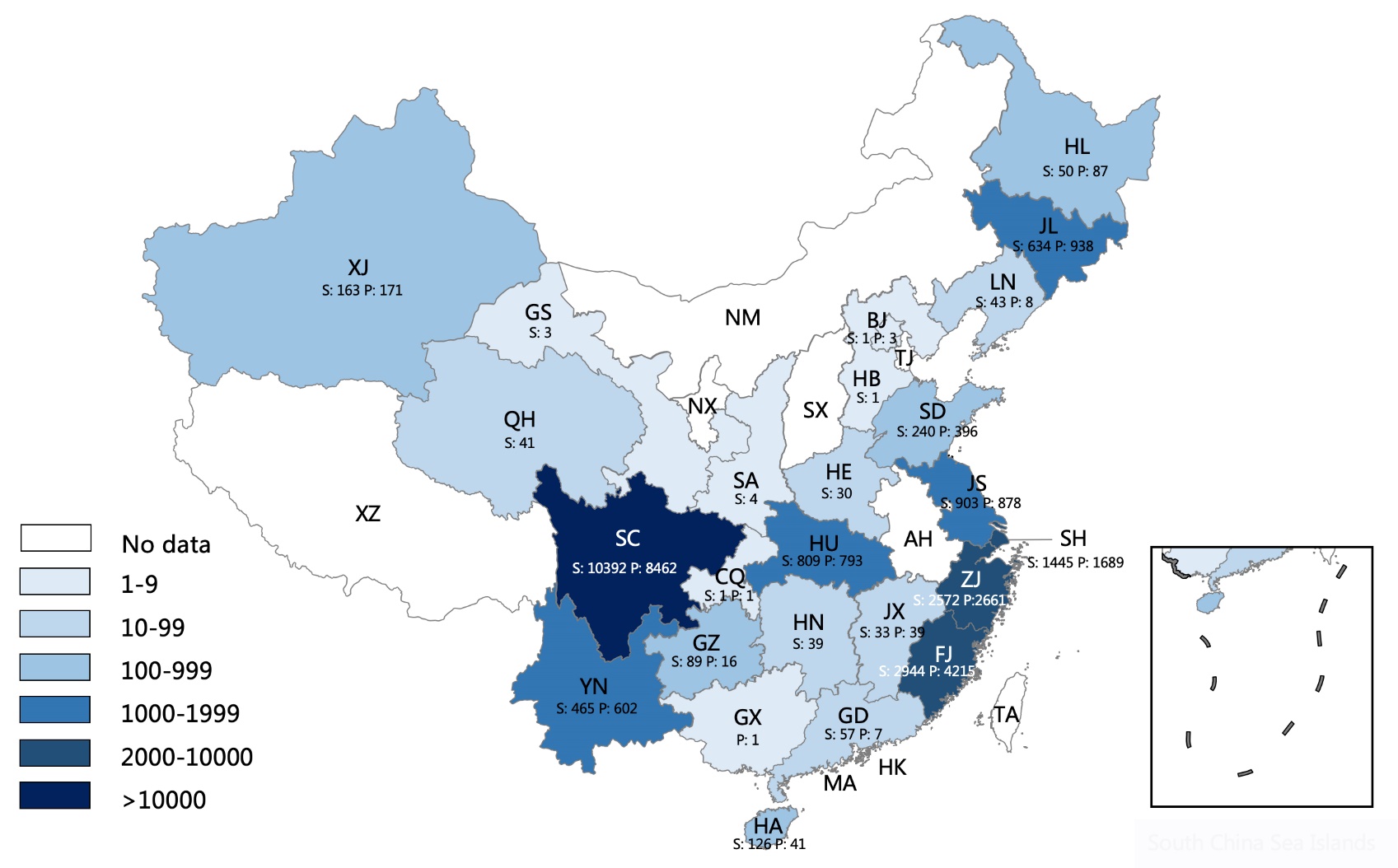
**

**Supplementary Figure 1. Geographic distribution of participants across China.** Abbrev: S: No. of students; P: No. of parents; AH, Anhui; BJ, Beijing; CQ, Chongqing; FJ, Fujian; GD, Guangdong; GS, Gansu; GX, Guangxi; GZ, Guizhou; HA, Hainan; HB, Hebei; HE, Henan; HK, Hong Kong; HL, Heilongjiang; HN, Hunan; HU, Hubei; JL, Jilin; JS, Jiangsu; LN, Liaoning; MA, Macao; NM, Inner Mongolia; NX, Ningxia; QH, Qinghai; SA, Shaanxi; SC, Sichuan; SD, Shandong; SH, Shanghai; SX, Shanxi; TA, Taiwan; TJ, Tianjin; XJ, Xinjiang; XZ, Tibet; YN, Yunnan; ZJ, Zhejiang; JX, Jiangxi.

**
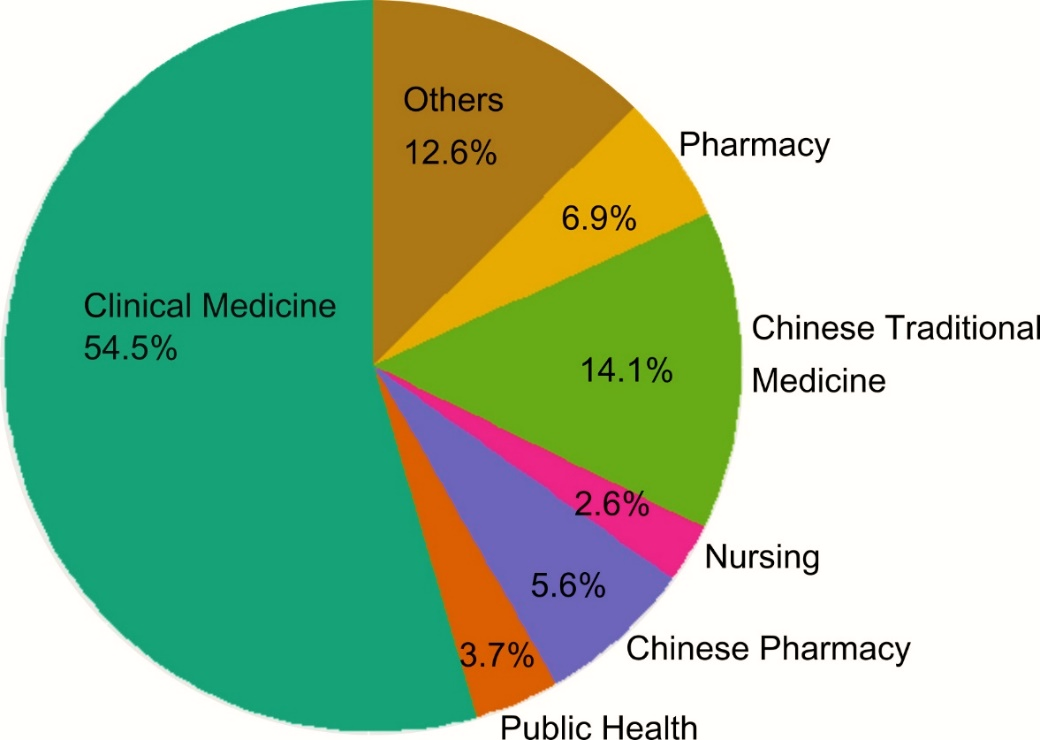
**

**Supplementary Figure 2.** The ranking of medical-related majors preferred by students.
